# Unmasking the Mimic: Radionecrotic Lesion Masquerading as Brain Neoplasia on Magnetic Resonance Imaging

**DOI:** 10.7759/cureus.59259

**Published:** 2024-04-29

**Authors:** Diego A. Barrios-González, Jimena Gonzalez-Salido, Jimena Colado-Martínez, Santiago Philibert-Rosas, Fernando Sotelo-Díaz, Mario A. Sebastián-Díaz, L. Jimena Gómez-Rodríguez, Nora E. Kerik-Rotenberg, Guillermo A. Gutiérrez-Aceves, Iris E. Martínez-Juárez.

**Affiliations:** 1 Epilepsy Clinic, National Institute of Neurology and Neurosurgery, Mexico City, MEX; 2 Epilepsy Clinic, National Institute of Neurology and Neurosurgery, Mexico City, Mexico, Mexico City, MEX; 3 Neurosurgery Residency Program, National Institute of Neurology and Neurosurgery, Mexico, MEX; 4 Department of Nephrology, South Central High Specialty Hospital PEMEX, Mexico, MEX; 5 Internal Medicine Residency Program, Hospital Médica Sur, Mexico City, MEX; 6 Molecular imaging unit, National Institute of Neurology and Neurosurgery, Mexico City , MEX; 7 Radioneurosurgery Unit, National Institute of Neurology and Neurosurgery, Mexico City, MEX

**Keywords:** tumor´, magnetic resonance imaging, cns neoplasia, radionecrosis, radiosurgical callosotomy

## Abstract

Corpus callosotomy is a therapeutic approach for drug-resistant epilepsy, with positive outcomes observed in managing atonic seizures. Despite a decline in its usage, radiosurgical callosotomy remains a viable option for drug-resistant epilepsy due to its low risks of post-radiation neoplasia, albeit not with exceptions. Brain radionecrosis is characterized by tissue death and vascular endothelial damage following the procedure. Despite the low risk of intracranial secondary malignancy associated with radiation in some cases, post-radiation lesions might present with distinct characteristics needing a thorough diagnostic approach. Herein, we present a unique case of a patient with focal epilepsy who developed a radionecrotic lesion following radiosurgical callosotomy, affecting the anterior cingulate cortex, and mimicking a central nervous system (CNS) tumor. Molecular imaging techniques, including 18-fluorodeoxyglucose positron emission tomography/computed tomography (18-FDG PET/CT) and 11C-acetate PET/CT scans, were employed to differentiate the lesion from a tumor. This case underscores the importance of considering radionecrosis as a differential diagnosis in patients who undergo radiosurgical callosotomy presenting with ring-like enhancement lesions on magnetic resonance imaging (MRI).

## Introduction

Since the correlation between the integrity of the corpus callosum and seizure frequency was established [1], corpus callosotomy (CC) has emerged as a therapeutic approach for managing drug-resistant epilepsy, notably yielding positive outcomes in decreasing atonic seizures [2]. Research indicates that radiosurgical CC produces comparable results to open surgery in seizure improvement [3], despite a decline in its usage due to radiation-related side effects. However, radiosurgical callosotomy remains a viable option for drug-resistant epilepsy based on the discretion of neurosurgeons [4] and the low risk of post-radiation neoplasia documented in the literature [5]. 

Although reports on radiosurgical CC in epilepsy are limited, they have contributed to understanding the clinical and radiological response following CC radiation. Consequently, it is anticipated that after one year post-CC, radionecrotic changes will be observed in the radiation target volume, unrelated to seizure improvement or complication emergence. Nonetheless, in some instances, the radionecrotic lesion may extend beyond the intended target volume [6]. 

There is evidence that radiosurgical CC has identical results in improving seizure control in drug-resistant epilepsy as open surgery [3]; however, its use has decreased due to the side effects of radiation exposure, although low risk of post-radiation CNS neoplasia after this procedure has been reported [5].

This report outlines a patient with focal epilepsy who underwent radiosurgical anterior CC with volumetric modulated arc therapy (VMAT) and developed a ring-like enhancing radionecrotic lesion on contrast magnetic resonance imaging (MRI). This lesion extended to the anterior cingulum and septum pellucidum, mimicking a central nervous system (CNS) neoplasia.

## Case presentation

A 22-year-old female, with a past medical history significant for a decompressive craniectomy secondary to subdural hematoma due to vitamin K deficiency at one month of age, subsequently developed cerebral palsy with right hemiparesis and a gross motor function scale of I.

Her epilepsy onset was at 10 years of age, marked by clusters of focal to bilateral tonic-clonic seizures occurring two to three times within 24 hours, prompting immediate initiation of antiseizure drugs (ASDs). During epilepsy, the patient also had right-sided focal clonic and hemiclonic seizures with preserved awareness, occurring at a frequency of 20 seizures per month, primarily during sleep. In addition, she presented atonic and focal seizures with automatisms of the right hand, averaging four occurrences per day, with a catamenial pattern observed in seizure presentation. 

The patient’s electroencephalogram (EEG) showed moderate-to-severe generalized slowing characterized by 5-7 Hz theta rhythm activity during wakefulness, along with epileptic activity consisting of bifrontal paroxysms of 1-2 Hz rhythmic slow spike and wave, as well as polyspike waves. 

**Figure 1:**
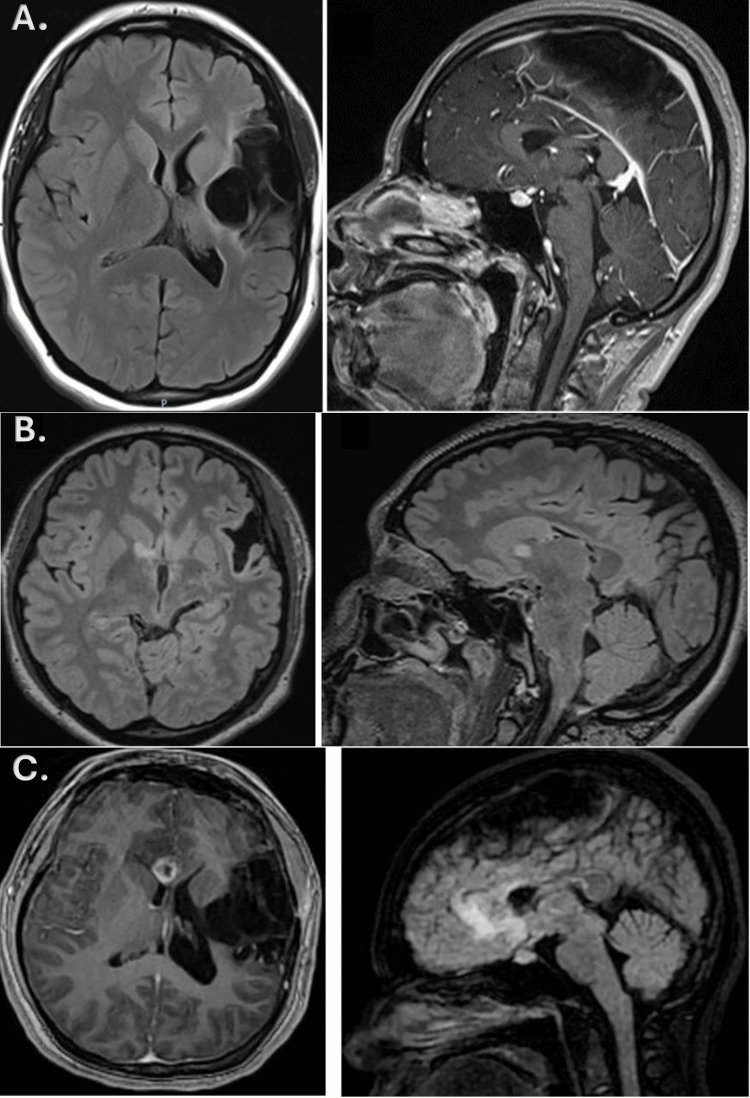
MRI findings. (A) Axial FLAIR and sagittal contrast-enhanced T1 sequences. A hypointense left frontoparietal cystic lesion can be observed, with an irregular contour and gliosis in the adjacent parenchyma. The lesion was diagnosed in childhood and caused an increase in the amplitude and retraction of the left lateral ventricle and the adjacent gyri. There was no evidence of any other structural alteration. (B) MRI findings three months after callosotomy. Axial and sagittal FLAIR sequences, with increased signal intensity at the level of the anterior arm and the knee of the right internal capsule. (C) Axial T1-contrast image showing ring-like enhancement lesions at the level of the knee of the residual corpus callosum and septum pellucidum, along with a sagittal FLAIR sequence displaying hyperintensity in the anterior cingulate cortex. FLAIR, fluid-attenuated inversion recovery; MRI, magnetic resonance imaging

Over time, a left frontoparietal arachnoid brain cyst was identified in a brain MRI, leading to a diagnosis of focal epilepsy for which she was referred to the National Institute of Neurology and Neurosurgery Mexico (NINN) (Figure 1A). She was put on ASD treatment consistent with levetiracetam (LEV) 500 mg/bid, oxcarbazepine (OXC) 200 mg, phenytoin (PTH) 100 mg/tid, and topiramate (TPM) 100 mg/bid with poor seizure response; at some point, she was also on ketogenic diet with no improvement in seizures. 

**Figure 2:**
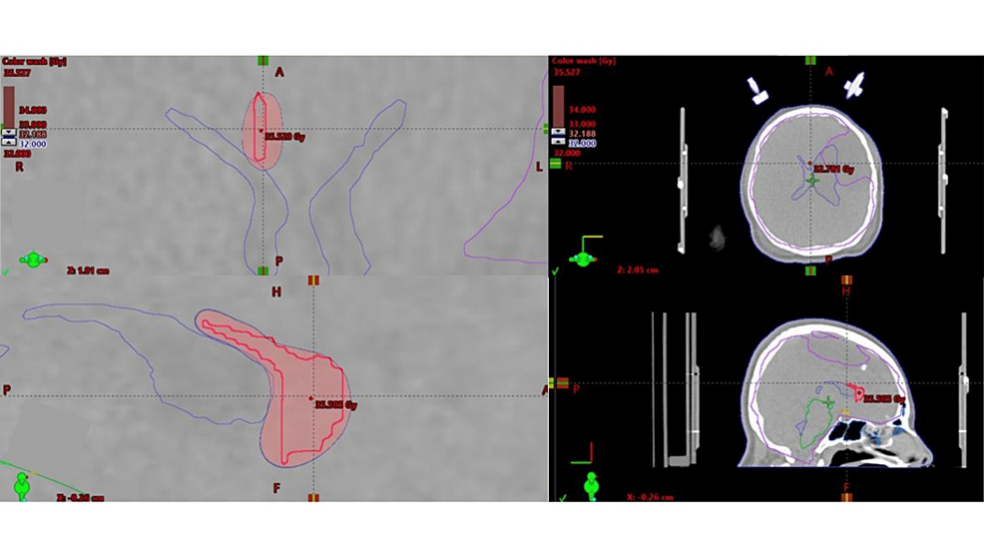
Radiosurgery treatment plan for anterior two-thirds callosotomy using a linear accelerator and VMAT technique. Images were obtained from the External Beam Planning Department of Radio Neurosurgery of the National Institute of Neurology and Neurosurgery, CDMX, Mexico. Tomography with a stereotactic radiation treatment plan in axial and sagittal views where the isodose curves in the treatment volume are observed: The blue line corresponds to the prescribed dose of 32 Gy to the periphery; the red line is the treatment volume. A maximum dose point (redpoint) of 35.5 Gy is also observed. VMAT, volumetric modulated arc therapy

Due to her drug-resistant epilepsy, during the epilepsy rounds, it was determined that radiosurgical CC represented a favorable therapeutic option for her condition. This course of treatment was then proposed to the patient. Radiosurgery of the anterior two-thirds of the corpus callosum was performed in the patient with a single dose of 32 Gray (Gy). The VMAT technique, facilitated by a LINAC® linear accelerator, was utilized for this procedure (Figure 2).

Three months after radiosurgical CC, the atonic seizures disappeared, and the seizure frequency decreased, with some persistent focal and focal to bilateral tonic-clonic seizures. The control MRI showed hyperintensity at the level of the anterior arm and knee of the right internal capsule with a slight ring enhancement at the same level. These changes were attributed to the radiological response after the procedure (Figure 1B).

Due to the epidemiological situation of the COVID-19 pandemic, the patient follow-up was interrupted for over two and a half years. Once the patient returned for follow-up, she and her mother reported an increased seizure frequency of up to 30 per month with the same semiology, in addition to the presence of apathy and irritability, consistent with a frontal lobe syndrome involving the anterior cingulate. 

A new contrast MRI showed a lesion with ring-like enhancement in the residual corpus callosum extending to the septum pellucidum and anterior cingulate gyrus (Figure 1C). Given these findings, a frontal lobe syndrome involving the anterior cingulate was diagnosed, and positron emission tomography (PET) was performed to rule out CNS tumor versus radionecrosis. Molecular imaging was performed using an 18-FDG radiotracer, and no hypermetabolism was identified at the lesion.

**Figure 3:**
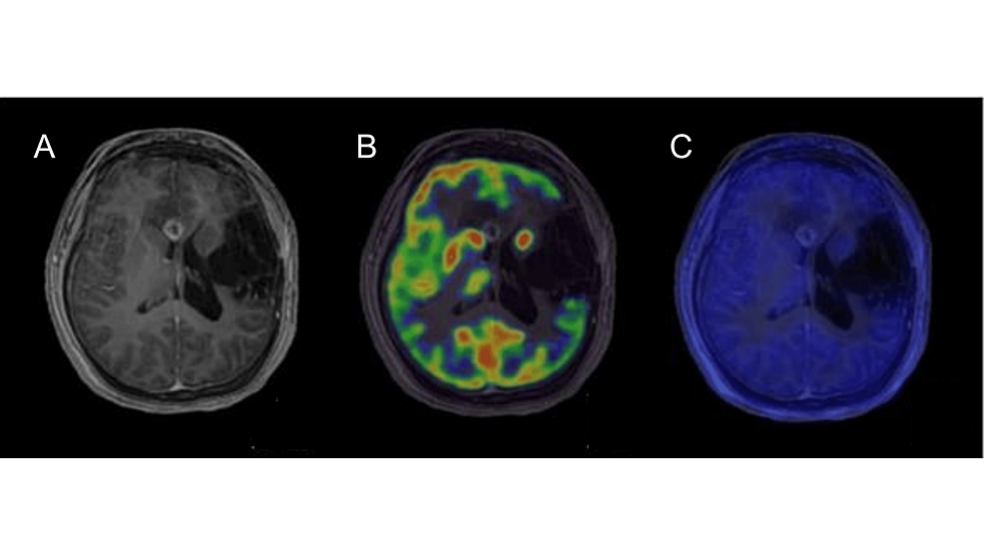
PET findings confirmed the presence of radionecrosis. (A) Located at the corpus callosum knee and was not associated with metabolism. (B) Positron emission tomography-fluorodeoxyglucose (PET-FDG) imaging with postsurgical changes at the level of the corpus callosum showed an ill-defined hyperdense image in its genual portion not associated with metabolism. Fused PET/MRI demonstrated the lesion in the previous MRI. (C) PET-acetate imaging with MRI fusion showed that the lesion did not present an increase in the transport and synthesis of fatty acids.

However, due to the history of radiotherapy and to rule out a low-grade glioma being masked by cortical metabolism in 18-FDG PET, another molecular imaging study with 11-Acetate-Carbon radiotracer was performed without evidence of neoplasia (Figures 3A-3C).

The lack of hypermetabolism in 18-FDG PET and increased fatty acid synthesis in 11-Acetate-Carbon PET were consistent with radionecrosis. Therefore, the patient was started on prednisone 60 mg per day for two months to decrease brain edema and improve seizures and personality changes. At the last follow-up, the seizure frequency had improved, atonic seizures had disappeared, and there was a moderate improvement in behavior.

## Discussion

In this study, we present a case of a patient with focal epilepsy who developed a radionecrotic lesion as a result of radiosurgical callosotomy. This lesion mimicked a CNS tumor and affected the anterior cingulate cortex, as evidenced by a ring-like enhancement lesion in addition to hyperintensities at the level of septum pellucidum and residual corpus callosum found on the patient's contrast MRI. The condition led to a frontal syndrome characterized by apathy and irritability.

To approach the lesion identified on the patient's contrast-enhanced MRI, molecular imaging techniques were employed. Initially, an 18-FDG PET/CT scan was conducted to eliminate the possibility of high-grade glioma. Subsequently, an 11C-acetate PET/CT scan was performed to rule out the presence of a low-grade glioma. This decision was based on its capability to further distinguish between grade III and grade IV neoplasms, as demonstrated in a study by Kim et al. [7]. 

Brain radionecrosis is tissue death caused by radiation, and it may follow radiotherapy to all or parts of the brain. It is most commonly observed after high-dose radiation treatment [8]. Moreover, it is thought to be part of late brain injury and is characterized by a combination of vascular endothelial damage and demyelination lesions, followed by neuronal death, and often does not recede [9]. 

Radiosurgical callosotomy is indicated for the treatment of *drop attacks* and generalized tonic-clonic seizures, and among the complications described for the procedure are edema, transient headache, and reversible neurologic deficit [6]. No higher risk of malignancy due to radiation exposure during radiosurgical CC has been reported [10]. Specifically, the risk of intracranial secondary malignancy in patients treated with stereotactic radiosurgery remains low at long-term follow-up [5] due to its high radiation doses and consequent absence of residual DNA in the target zone, compared to low-dose radiotherapy. The latter promotes genomic instability through DNA double-strand breaks in DNA residual [11].

This information, along with the clinical, radiological, and molecular imaging characteristics of the lesion, helped guide the diagnosis of our patient's case, which is deemed to be a lesion corresponding to radionecrosis secondary to callosotomy. To our knowledge, this is the first report of radionecrotic lesion with ring-like enhancement and a diagnostic approach to rule out CNS neoplasia after radiosurgical CC.

## Conclusions

Performing CC via radiosurgery has both advantages and potential risks. While it can effectively treat certain seizure types and syndromes, there's a risk of radionecrosis, which may mimic CNS tumors. Therefore, it is essential to exercise caution during radiosurgery follow-up MRIs to prevent misdiagnosis and unnecessary procedures such as brain biopsies. This caution is particularly crucial in regions with limited alternative treatments like vagus nerve stimulation (VNS) or deep brain stimulation (DBS).

Vigilant monitoring of both the procedure's short- and long-term consequences is crucial to safeguard patients' quality of life and prevent unnecessary interventions. Moreover, it is important to raise awareness about the possibility of scars resulting from radiosurgery resembling CNS neoplasia, emphasizing the necessity for thorough evaluation and management.
